# Incidence and Risk Factors of Ventilator-Associated Pneumonia in Cardiac Arrest in Patients With Selective Digestive Decontamination

**DOI:** 10.1155/ccrp/7669466

**Published:** 2025-03-26

**Authors:** Erik Roman-Pognuz, Stefano Di Bella, Alberto Enrico Maraolo, Mauro Giuffrè, Chiara Robba, Giuseppe Ristagno, Clifton W. Callaway, Umberto Lucangelo

**Affiliations:** ^1^Department of Medical Science, University of Trieste, Trieste, Italy; ^2^Department of Clinical Medicine and Surgery, Section of Infectious Disease, University of Naples “Federico II”, Naples, Italy; ^3^Yale University, New Haven, Connecticut, USA; ^4^Department of Surgical and Diagnostic Science, University of Genova, Genoa, Italy; ^5^Department of Medical-Surgical Physiopathology and Transplants, University of Milan, Milan, Italy; ^6^Department of Emergency Medicine, University of Pittsburgh, Pittsburgh, Pennsylvania, USA

**Keywords:** antibiotics, cardiac arrest, hypothermia, intensive care, pneumonia, selective digestive decontamination, targeted temperature management, temperature

## Abstract

**Background:** Out-of-hospital cardiac arrest (OHCA) is a leading cause of morbidity and mortality. Temperature management (TM) is recommended since hyperthermia is associated with worse outcomes. Pneumonia is a frequent occurrence following OHCA, and some studies suggest that TM may have a negative impact on its development. Selective digestive decontamination (SDD) is used in some centers to reduce the incidence of pneumonia in intensive care unit (ICU), but its use remains controversial. This study aims to assess the incidence, risk factors and clinical course of VAP after OHCA.

**Methods:** We conducted a retrospective cohort study on 169 consecutive OHCA patients after their admission in ICU. All patients were treated with TM and SDD. Pharyngeal swabs were analyzed twice weekly. The primary outcome was the incidence of VAP and non-VAP. Secondary aim was to identify the risk factors associated with VAP and its effect on patients' outcome.

**Results:** Incidence of VAP was 5.3%, while incidence of non-VAP was 9.5%. In multivariate analysis, male gender (sHR 3.01; CI 1.1–7.9), increase of white blood cells (WBC) count > 30% over 5 days (sHR 2.32; CI 1.23–3.9), heart disease (sHR 2.4; CI 1.36–4.59), and bacterial colonization of the pharynx (sHR 2.79; CI 1.13–4.39) were significantly associated with VAP.

**Conclusions:** Pharyngeal colonization could be useful to identify patients at higher risk of VAP development. The low rate of VAP in this cohort suggests that SDD can prevent VAP after OHCA. Further studies are needed to explore the potential of SDD in OHCA patients.

## 1. Introduction

Cardiac arrest is one of the leading causes of death and disability worldwide, with overall survival rates after out-of-hospital cardiac arrest (OHCA) not exceeding 10%–20% despite advancements in postresuscitation care [1, 2]. During resuscitation, the lungs are exposed to risks such as pulmonary contusions and aspiration, which increase the likelihood of respiratory complications [3, 4]. Moreover, postcardiac arrest syndrome (PCAS) due to ischemia-reperfusion injury alters immunological responses and may elevate the risk of systemic infections [5]. In these patients, temperature management (TM) is recommended to reduce neurological damage and improve outcomes [2, 6]. However, TM, particularly when using hypothermia, has been associated with increased rates of infections, including early-onset pneumonia (EOP) [7, 8]. TM at temperatures below 36°C can impair immune responses by reducing leukocyte function and cytokine production, which may exacerbate the risk of infections in the early stages of ICU admission [9, 10]. Some studies have reported a higher incidence of pneumonia in patients managed with hypothermia compared to those managed with normothermia [11, 12].

The role of antibiotic prophylaxis remains unclear [13]. However, studies, including a large German cohort, suggest antibiotic prophylaxis may reduce infections associated with prolonged mechanical ventilation and ICU stays, significantly decreasing pneumonia and sepsis rates [14, 15]. Selective Digestive Decontamination (SDD) is an infection prevention strategy designed to reduce oropharyngeal and gastrointestinal colonization by potentially pathogenic microorganisms, thereby lowering the incidence of respiratory infections and ventilator-associated pneumonia (VAP). The SDD protocol typically involves the use of nonabsorbable antibiotics applied to the oropharynx and gastrointestinal tract, combined with short-term systemic antibiotics. SDD reduces VAP rates among general ICU patients by targeting Gram-negative bacteria, which are common causes of hospital-acquired infections.

The role of SDD in post-OHCA patients undergoing TM remains underexplored. Notably, previous studies have reported reduced VAP incidence with SDD, though the persistent presence of multidrug resistant organisms (MDROs) in certain cases suggests limitations in the scope of its protective effects [16, 17]. Another recent multicenter trial on OHCA patients with shockable rhythms found that 2-day antibiotic prophylaxis with amoxicillin/clavulanate reduced early-onset VAP incidence (9% vs. 34%) but did not affect ventilator-free days or 28-day mortality [1]. Despite these findings, the true incidence of VAP remains uncertain, with reports of up to 25% in other mechanically ventilated patient populations [18].

This retrospective study investigates the incidence of VAP and non-VAP in post-OHCA patients treated with TM and SDD, with a focus on evaluating the potential role of SDD as a prophylactic measure to reduce pneumonia rates. Identifying a subgroup of patients who might benefit the most from a prophylactic antibiotic protocol could be useful to enhance risk stratification and inform prophylactic planning, but clear risk factors for predicting at admission the development of pneumonia have not yet been identified [11, 19] Therefore, this study also seeks to identify whether pharyngeal bacterial colonization and baseline patient characteristics are independent risk factors for pneumonia following cardiac arrest.

## 2. Methods

### 2.1. Patients and Data Collection

This retrospective cohort study was conducted in a 16-bed ICU at Trieste University Hospital, Italy, and included all consecutive patients admitted after OHCA from February 2012 to June 2019. Patients were intubated at the scene and mechanically ventilated upon ICU admission. Based on individual patient factors, such as no-flow time, return of spontaneous circulation (ROSC) time, and reactive pupil response, Targeted TM was customized to either 33°C or 36°C. Routine VAP prevention measures, including head-of-bed elevation and daily oral care, were applied consistently across all patients.

### 2.2. SDD Protocol

According to our institution's infection control practices, all patients received the SDD protocol from the day of ICU admission. The SDD regimen consisted of the following:

1. Oral Paste: Applied every 6 h to the oral cavity by ICU nursing staff, the paste contained 2% polymyxin E, 2% tobramycin, and 2% amphotericin B in a 1 g dosage. For patients with methicillin-resistant *Staphylococcus aureus* (MRSA) colonization, 2% vancomycin was added to the paste.
2. Nasogastric Administration: The nasogastric antibiotic suspension included 100 mg of polymyxin E, 80 mg of tobramycin, and 500 mg of amphotericin B, administered every 6 h.
3. Systemic Antibiotic: Intravenous cefotaxime (6 g per day as a continuous infusion) was given for the first 5 days, targeting a broad range of Gram-negative and some Gram-positive bacteria.

SDD protocol with oral paste and nasogastric antibiotics continued until the patient was extubated, while systemic cefotaxime administration was limited to the initial 5 days. Oropharyngeal swabs were obtained upon admission and biweekly thereafter to monitor pharyngeal colonization and detect potential MDROs.

Data were collected retrospectively from a database in use in our Institution to monitor the infections and the prevalence of MDR microorganisms in ICU. C-reactive protein (CRP) and white blood cells (WBC) were assessed every day, while WBC differential was evaluated on admission and then twice a week. The diagnosis of VAP was based on clinical and microbiological criteria: a new or progressive infiltrate on chest radiographic examination and at least one of the following clinical symptoms: fever > 38°C, leukocytosis at least 12,000 cells/μL, leukopenia less than 4000 cells/μL, and purulent tracheobronchial secretions [20–22]. The study was approved by the local Ethic Committee (protocol n. 133_2022H).

### 2.3. Outcomes

The primary outcome measure of the study was the development of VAP after ICU admission, defined as a time-to-event endpoint. Patients were followed until ICU discharge or death, whichever occurred first.

As secondary outcomes, the study examined the associations between demographic characteristics, previous medical history, laboratory test results, fungal and bacterial pharyngeal colonization, and the occurrence of VAP or other types of pneumonia (aspiration and CAP) after ICU admission.

### 2.4. Definitions of VAP and Non-VAP

VAP and non-VAP were defined using established radiographic, systemic and pulmonary criteria [22, 23]: To differentiate between types of pneumonia in this study, we use the following definitions:

• VAP: Pneumonia that develops after 48 h of ICU admission in patients who are mechanically ventilated. Diagnosis of VAP is based on clinical, radiological, and microbiological criteria, including new or progressive infiltrates, consolidation or cavitation on chest radiographs, fever > 38°C, leukocytosis (> 12,000 cells/μL) or leukopenia (< 4000 cells/μL), and purulent respiratory secretions.
• Non-VAP: Pneumonia that develops within the first 48 h of ICU admission. This classification includes both aspiration and community-acquired pneumonia, likely arising from events surrounding the cardiac arrest and resuscitation process or from prior colonization.

### 2.5. Rationale for Cutoff Values

Clinically significant increases for WBC count and CRP were defined based on their clinical relevance and our institution's historical data. An increase of > 30% in WBC and CRP levels over the initial 5 days of ICU stay was chosen as a marker for significant systemic inflammation. These specific thresholds align with our institution's infection surveillance protocols used to detect early signs of infection in critically ill patients.

### 2.6. Statistical Analysis

Descriptive statistics included median and interquartile range (IQR) for continuous variables and proportions for dichotomous and categorical variables. Categorical variables were compared with the Chi-square test, while continuous variables were compared with the *t*-test or Mann–Whitney test as appropriate.

The study analyzed the time-to-first event using survival analysis for VAP as the primary outcome, and any pneumonia as the secondary outcome. However, death is a competing risk and relying solely on the Kaplan–Meier estimate of the survival function could lead to potentially biased results in the estimation of the incidence function. [24]. Therefore, a competing risk model investigated the effect of the exposure of interest on the outcome incidence. Firstly, we estimated the cumulative incidence function (CIF) over the 88-months study period. Secondly, the association between this variable and time-to-first event was gauged through multivariable Fine and Gray's regression models for proportional hazard, that give estimates of the subdistribution hazard ratio (sHR) (i.e., the relative change in the instantaneous rate of occurrence of the given type of event in subjects who have not yet experienced an event of that type) [24, 25] and its associated 95% CI. Specifically, two Fine and Gray's models were built to regress the sHR of VAP (Model 1) and any pneumonia (Model 2). The exposure of interest was adjusted for the covariates with proven univariable-model significance among a set of potential candidates selected based on expert knowledge. All analyses were carried out using the statistical software R, version 4.0.5 Version (R Core Team, 2021), using the “cmprsk” package (version 2.2-10). A two-tailed *p* value < 0.05 was deemed statistically significant.

### 2.7. Sample Size and Power Calculation

This retrospective study used all available data, and we did not perform an a priori sample size calculation. However, our cohort of 169 patients provides a substantial sample for evaluating the incidence of VAP and non-VAP in post-OHCA patients treated with TM and SDD.

## 3. Results

Data from 169 subjects admitted to an ICU for 7 years were analyzed. 5.3% of patients developed VAP, while 9.5% developed other kinds of pneumonia.  Table 1 describes the baseline features and outcomes for the population. Patients were mostly men (71.6%); with the largest proportion (27.2%) in age groups 60–69 years and 70–79 years. The most common comorbidity was hypertension (39%), followed by chronic heart disease (33.1%) and diabetes (13%). The median value of the Simplified Acute Physiology Score 2 (SAPS2) was 58 (IQR 45–67). Pharyngeal colonization by yeast was present in 34.9% of cases, by bacteria in 10.6%. A total of 69 patients (39.6%) died in the hospital, with a median time to death of 6 days (IQR 3–11). In total, 9 patients developed VAP (5.3%), 16 subjects developed non-VAP (9.5%).

Table 2 shows the univariate and multivariate analysis of the factors predictive of VAP. In univariate analysis, the following variables were associated with VAP: male gender, increase of WBC count over 30% in 5 days, ventilation duration, SAPS2, heart disease, diabetes, pharyngeal colonization by a yeast, or a bacterium. In multivariate analysis including variables found to be statistically significant in the univariate analysis, the following variables were independently associated with VAP: male gender (sHR 3.01, 95% CI 1.10–7.9), increase of WBC count > 30% over 5 days (sHR 2.32, 95% CI 1.23–3.9), heart disease (sHR 2.40, 95% CI 1.36–4.59), pharyngeal colonization by a bacterium (sHR 2.79, 95% CI 1.13–4.39).

Table 3 illustrates the univariate and multivariate analysis of the predictors of non-VAP. In univariate analysis, the following variables were associated with non-VAP: gender male, increase in CRP > 30% in 5 days, increase of WBC count over 30% in 5 days, ventilation days, SAPS2, cancer, bacterial colonization of the pharynx. In multivariable analysis, variables independently associated with non-VAP were: male gender (sHR 1.76, 95% CI 1.0–3.09), increase of WBC count > 30% over 5 days (sHR 3.25, 95% CI 1.8–5.6), pharyngeal colonization by a bacterium (sHR 1.67, 95% CI 1.01–2.76).

Table 4 describes the species of microorganisms isolated from either tracheo-bronchial aspirate or bronchoalveolar lavage. In 7 patients of the “VAP” group, cultures identified 7 bacteria. Of these, 4 (57.1%) were MDR: 3 MRSA and 1 AMPc β lactamase producing *Escherichia coli.* Cultures from the other 3 patients with VAP revealed a sensible bacteria strain: 1 *Enterococcus faecalis* and 2 methicillin susceptible *Staphylococcus aureus* (MSSA). Cultures in 10 patients of the “other pneumonia” group identified: 1 *Enterococcus faecalis*, 1 MSSAMSSA, 2 *Pseudomonas aeruginosa*, 1 *Enterobacter aerogenes*, 2 *Heamophilus influenzae*, 2 *Escherichia coli* and 1 *influenza A* H1N1.

**Figure 1:**
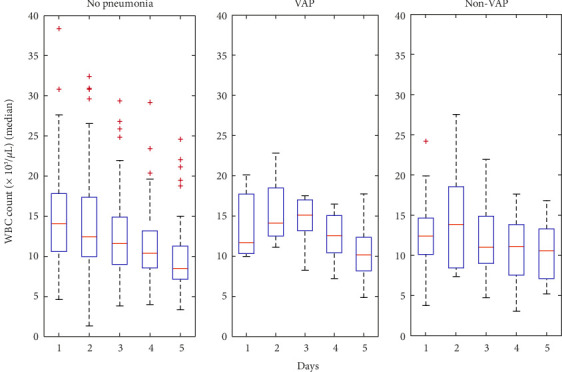
WBC count (× 10^3^/μL) (median) during the first 5 days of ICU stay in “no pneumonia” group, “VAP” group, and “non-VAP” group. A > 30% increase of WBC count in VAP patients was greater than the modest increase ini non-VAP patients and minimal changes in those without pneumonia.

**Figure 2:**
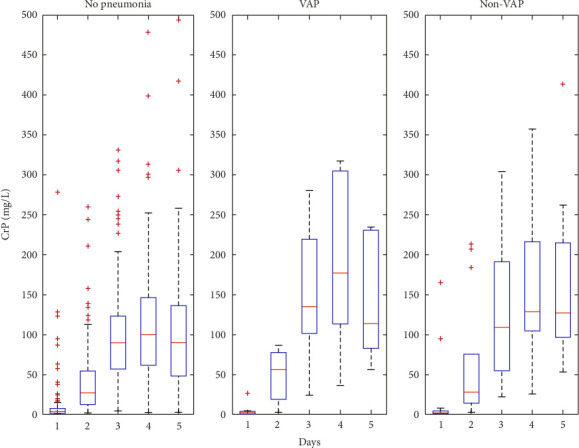
CRP (mg/L) (median) during the first 5 days of ICU stay in stay in “no pneumonia” group, “VAP” group, and non-VAP group. CRP levels in VAP patients exhibited larger increase over the first 5 days than in the non-VAP group or in patients without pneumonia.

The WBC and CRP data indicate heightened inflammation among patients with pneumonia.  Figure 1 shows WBC counts in VAP patients increased > 30% over the first 3 days. This increase in WBC count was associated with VAP in both univariate and multivariate analysis (Table 3).  Figure 2 shows that CRP levels in VAP patients increase over the first 5 days, reflecting a strong systemic inflammatory response. This increase in CRP aligns with the WBC data, reinforcing the potential value of these markers as early indicators of infection. In the univariate analysis, an association was found between an increase of CRP > 30% and the “VAP” group (Table 3), while there was no statistically significant association in the “other pneumonia group”.

## 4. Discussion

VAP is a common complication in comatose patients admitted to the ICU after OHCA and treated with TM. This study provides a unique perspective on the use of SDD in preventing VAP and non-VAP in a post-OHCA population managed with Targeted TM. While several studies have explored the use of SDD in ICU patients to reduce VAP incidence, relatively few have focused specifically on OHCA patients undergoing TM, a group at elevated risk for infectious complications due to immunosuppressive effects of hypothermia and prolonged mechanical ventilation. In our cohort, we found that VAP occurred in 5.3% of patients, while 9.5% developed other pneumonia types, a rate that is lower compared to the incidence rates reported in other OHCA patient cohorts [1, 16, 26].

In addition, our study uniquely highlights the persistence of MDROs in VAP cases, suggesting that while SDD may reduce overall VAP incidence, its efficacy may be limited when MDROs are prevalent. For clarity and to differentiate this study's contributions, Table 5 summarizes findings from relevant prior studies and contrasts them with our results.

In our study, OHCA patients were treated with TM at 33°C and received oral decontamination with chlorhexidine 2%. About 39% of the patients received antibiotics, mostly beta-lactams, within the first 7 days of ICU admission, with an average delay of 2.17 days. Similar VAP incidences were reported in previous studies: Scamperle et al. reported 5.7% incidence with therapeutic hypothermia and SDD protocol, while Manchal et al. found a 6% incidence without SDD or antibiotic prophylaxis ([17, 27]). Our study also observed a 9.5% incidence of non-VAP, possibly due to macro or micro aspirations during resuscitation or pre-existing lung infections.

### 4.1. SDD and Non-MDRO VAP Prevention

Our data support the premise that SDD can be effective in reducing non-MDRO VAP by minimizing colonization with susceptible Gram-negative bacteria, such as *Klebsiella* and *E. coli*. In our study cohort, a majority of the non-VAP cases involved organisms that were either susceptible to cefotaxime or were not MDR. The prophylactic effects of SDD are likely due to its ability to selectively target Gram-negative organisms, which commonly colonize the oropharyngeal and gastrointestinal tracts and are frequent causative agents in hospital-acquired infections.

However, SDD's limitations become apparent in cases involving MDROs, which constituted a significant portion of the VAP cases in our cohort. The persistence of MDROs, such as MRSA and AmpC β-lactamase-producing *E. coli*, in VAP cases suggests that SDD alone may not provide sufficient coverage to prevent infections with these organisms. While SDD does target some Gram-negative bacteria, its inability to cover certain resistant strains, particularly Gram-positive pathogens, may explain the persistence of MDRO-related VAP in our patient population. This finding underscores the need to consider additional or alternative prophylactic strategies when MDRO colonization is suspected or identified.

In the present cohort, VAP developed on average after 5 days of ICU stay, when SDD is supposed to have already started working. Most bacterial strains found in the respiratory secretions of VAP patients were MDR. A possible interpretation of this finding could be that SDD is likely to have prevented pneumonia caused by susceptible bacteria, leaving only MDR bacteria to survive. Indeed, in our study the cultures of patients with VAP showed a high incidence of Gram-positive bacteria against which SDD prophylaxis is not active. The organisms associated with VAP vary according to many factors, but literature mainly reports a higher incidence of Gram-negative bacteria, while between the Gram-positive, the principal strain observed is *Staphylococcus aureus* [28]. In the group “other pneumonia” (non-VAP), lung infections developed approximately up to 48 h from admission were included, when cefotaxime in administered and SDD is likely not yet active. In this group, half of the bacteria strains were susceptible to cefotaxime and half were not. No MDR bacteria strains were found in the “other pneumonia” (non-VAP) group.

While the demographic characteristics of the patients were similar between the “VAP” group and the non-VAP group, the multivariate analysis identified male gender, heart disease, and pharyngeal colonization as independent risk factors for both VAP and non-VAP. These factors suggest a general susceptibility to pulmonary infections in this patient group rather than being exclusive to VAP. However, there may be underlying mechanisms linking these factors to VAP specifically.

1. Male Gender: Studies have indicated that males are generally more prone to pulmonary infections, possibly due to differences in immune response. This could relate to hormonal differences that impact the immune system, along with behavioral and cultural factors that influence health-seeking behaviors. In the context of VAP, male patients may experience prolonged mechanical ventilation and ICU stays, further increasing their infection risk.
2. Heart Disease: The association of heart disease with an increased risk of VAP is notable. Cardiovascular diseases can compromise pulmonary circulation, leading to impaired respiratory function and reduced clearance of respiratory pathogens. In our cohort, heart disease nearly doubled the risk of VAP, which may be attributed to an increased likelihood of impaired cardiovascular function complicating the body's response to respiratory pathogens.
3. Pharyngeal Colonization: Colonization of the pharynx by pathogenic bacteria was a significant risk factor for both VAP and non-VAP. This finding highlights the role of bacterial colonization as a precursor to respiratory infection, especially in mechanically ventilated patients. SDD's role in reducing VAP likely stems from its ability to reduce such colonization, although this effect may be limited to susceptible strains and less effective for MDROs.

The univariate analysis also showed a correlation between SAPS2 at admission and both VAP and other pneumonia. [29]. SAPS2 provides an estimate of the risk of death. The findings of this study suggest that a correlation exists between patient frailty and a higher likelihood of developing a lung infection. Ventilation length was significantly associated both with VAP and other pneumonia. It is known that the cumulative incidence is related to total duration of mechanical ventilation. On the other hand, the presence of pneumonia may increase the need of ventilation. This finding is confirmed by literature, it is also demonstrated that VAP may extend the length of ICU stay and increase the rate of tracheostomy [14].

A correlation between an increase of WBC > 30% during the first 5 days and VAP/other pneumonia was found both at univariate and multivariate analysis (Figure 1). Davies et al. in 2012 studied WBC trend in OHCA patients and did not find a specific pattern or discriminatory value for lung infection [16]. Hellenkamp et al. in 2015 did not find a correlation between WBC count in 3 measurement and the diagnosis of early pneumonia [14]. Ribaric et al. in 2016 also did not find found a correlation with WBC [30].

In the univariate analysis, a significant association was observed between an increase of CRP > 30% and the “VAP” group, while no significant association was found in the “other pneumonia group. Nonetheless, this association was not confirmed in the multivariate analysis. CRP is a quite nonspecific marker, which tends to increase in almost all patients in the first 3 days following OHCA. Oppert et al. demonstrated that the time course of CRP, in the patients with and without VAP were not significantly different, instead the time course of procalcitonin, differed between the groups [31]. Other studies found that procalcitonin, as CRP, is not discriminatory [32, 33]. In our cohort procalcitonin was not evaluated.

Our findings contribute to the growing body of literature on infection prevention strategies in postcardiac arrest care and underscore the need for further research in several areas:

1. SDD Efficacy in Different Patient Subpopulations: Future studies could explore whether specific subgroups, such as those with different cardiac comorbidities or varying immune responses, benefit more from SDD. This could help tailor SDD use to those who are most likely to benefit.
2. Alternative Prophylactic Strategies for MDROs: Given the limitations of SDD in preventing MDRO-related VAP, future research should investigate the effectiveness of alternative or supplementary strategies, such as selective oral decontamination with agents effective against Gram-positive bacteria or broader-spectrum prophylaxis approaches.
3. Long-term Outcomes of SDD in Post-OHCA Patients: Our study focused on short-term outcomes such as VAP incidence; however, future studies should assess the impact of SDD on long-term patient outcomes, including ICU length of stay, duration of mechanical ventilation, and overall survival. This will provide a more comprehensive picture of the potential benefits of SDD in ICU settings.

## 5. Limitations

The present study has some limitations, with the primary one being its retrospective nature as a single-center cohort study. The data were collected over a span of 7 years, during which certain aspects of standard care and the hospital's bacterial flora might have changed. For instance, patients in our cohort were treated with hypothermia or normothermia, future comatose patients admitted after OHCA will more often be treated only with normothermia due to the results of TTM2 trial [34], that may lead to a reduction in infectious complications. Additionally, our study assessed the incidence of VAP and revealed a lower rate compared to literature data. However, due to the study design, we were unable to evaluate long-term mortality, which could have provided valuable insights into the impact of VAP on survival. Additionally, eosinophils were only measured at two time points, and not all patients underwent cultures from tracheo-broncho aspirate or broncho-alveolar lavage [35–39]. Despite these limitations, the study successfully achieved its aim of assessing the association between patients' characteristics and the onset of VAP/other pneumonia.

## 6. Conclusions

The study identifies male gender, heart disease, and bacterial pharyngeal colonization as independent risk factors for VAP development in OHCA patients. The low VAP incidence in our cohort may be attributed to the routine application of SDD protocol regimens in our ICU. Further research is necessary to elucidate the role of antimicrobial prophylaxis and its potential benefits in specific patient subgroups. A randomized placebo-controlled multicenter trial would be instrumental in assessing the efficacy of the SDD protocol in reducing VAP among OHCA survivors.

## Figures and Tables

**Table 1:** Characteristics of the patients at the baseline.

Variable of interest	All subjects *n* = 169	No pneumonia *n* = 144 (85.2%)	VAP *n* = 9 (5.3%)	*p* value	Non-VAP *n* = 16 (9.5%)	*p* value
Gender, male	121 (71.6%)	100 (69.4%)	8 (88.9%)	0.21	12 (75%)	0.65
Age (years)						
Median (IQR)	66 (58; 75)	67 (58; 76)	67 (65; 74)		60 (50; 65)	
≤ 29	5 (2.96%)	5 (3.47%)	0	0.57	0	0.45
30–39	5 (2.96%)	3 (2.08%)	1 (11.1%)	0.10	1 (6.25%)	0.31
40–49	12 (7.1%)	9 (6.25%)	0	0.44	3 (18.75%)	0.07
50–59	28 (16.6%)	25 (17.36%)	0	0.17	3 (18.75%)	0.89
60–69	46 (27.2%)	36 (25%)	4 (44.4%)	0.20	6 (37.5%)	0.28
70–79	46 (27.2%)	41 (28.47%)	3 (33.3%)	0.75	2 (12.5%)	0.17
80–89	26 (15.4%)	24 (16.7%)	1 (11.1%)	0.66	1 (6.25%)	0.28
≥ 90	1 (0.6%)	1 (0.7%)	0	0.80	0	0.74
In-hospital death	67 (39.6%)	58 (40.3%)	4 (44.5%)	0.48	5 (31.3%)	0.80
Time before death (days)	6 (3; 11)	5 (2; 12)	21 (8; 36)		3 (3; 4)	
CRP at admission (mg/dL)	3.50	3.7	2.3	0.22	1.9	0.08
	(1.3; 7.4)	(1.4; 7.4)	(1; 3.6)		(1.2; 4)	
WBC at admission (cells/mm^3^)	13,950	14,060	11,600	0.70	12,410	0.19
	(10,450; 17,525)	(10,610; 17,760)	(10,300; 17,410)		(10,300; 14,630)	
Eosinophils percentage at admission	0.30 (0.61)	0.28 (0.60)	0.41 (0.61)	0.34	0.47 (0.74)	0.31
Days under ventilation	5 (3; 8)	5 (3; 8)	7 (5; 33)	0.03	5 (3; 7)	0.53
SAPS2 at admission	58 (45; 67)	57 (45; 65)	69 (57; 74)	0.11	62 (58; 75)	0.07
Comorbidities						
Hypertension	66 (39%)	60 (41.7%)	2 (22.2%)	0.25	4 (25%)	0.20
Chronic heart disease	56 (33.1%)	50 (34.7%)	5 (55.6%)	0.21	1 (6.2%)	0.02
Diabetes	22 (13%)	16 (11.1%)	2 (22.2%)	0.32	4 (25%)	0.11
Chronic lung disease	16 (9.5%)	16 (11.1%)	0	0.29	0	0.16
Obesity	10 (5.9%)	10 (6.9%)	0	0.41	0	0.28
Chronic kidney disease	10 (5.9%)	8 (5.5%)	1 (11.1%)	0.49	1 (6.2%)	0.91
Malignancy of any kind	7 (4.1%)	6 (4.1%)	1 (11.1%)	0.33	0	0.41
Pharyngeal yeast	59 (34.9%)	51 (35.4%)	4 (44.4%)	0.58	4 (25%)	0.41
Most represented species						
*Candida* spp	50 (84.7%)	42 (82.4%)	4 (100%)	0.33	4 (100%)	0.73
Pharyngeal bacteria	18 (10.6%)	12 (8.3%)	2 (22.2%)	0.16	4 (25%)	0.04
Most represented species						
*Klebsiella* spp	5 (27.7%)	3 (25%)	2 (100%)	0.001	0	0.56
*Escherichia coli*	5 (27.7%)	4 (33.3%)	0	0.61	1 (25%)	0.45

*Note:* Data are reported as median (IQR), mean (SD) or number (%).

**Table 2:** Competing risks analysis for the development of non-VAP.

	Univariate analysis		Multivariate analysis	
	sHR (95% C.I.)	*p* Value	sHR (95% C.I.)	*p* Value
Age	1 [0.98–1.02]	0.88		
Gender (male)	3.28 [1.29–8.32]	0.013	3.01 [1.10–7.9]	0.02
CRP at admission	0.96 [0.91–1]	0.25		
Increase of CRP > 30% in 5 days	1.78 [1.23–1.97]	0.002		
WBC at admission	0.97 [0.93–1.02]	0.28		
Increase of WBC > 30% in 5 days	1.92 [1.10–2.40]	0.001	2.32 [1.23–3.9]	0.04
Eosinophil % at admission	2.01 [0.90–2.7]	0.324		
Ventilation days	1.04 [1.02–1.05]	0.007		
SAPS2 at admission	1.03 [1.01–1.05]	0.0083		
Chronic lung disease	0.91 [0.80–1.10]	0.61		
Hypertension	0.72 [0.80–1.44]	0.23		
Heart disease	2.48 [1.39–4.45]	0.002	2.40 [1.36–4.59]	0.0001
Shockable rhythm	1.03 [0.90–1.10]	0.56		
Diabetes	1.9 [1.03–3.79]	0.069		
Obesity	1.42 [0.56–3.5]	0.63		
Kidney disease	1.71 [0.73–3.98]	0.44		
Cancer	3.34 [1.14–7.93]	0.006		
Pharyngeal yeast	1.47 [0.82–2.62]	0.19		
Pharyngeal bacteria	2.06 [1.03–4.11]	0.041	2.79 [1.13–4.39]	0.003

*Note:* For the multivariate analysis only the independently associated variables with sHR > 1 are reported.

**Table 3:** Competing Risks Analysis regarding the development of VAP.

	Univariate analysis		Multivariate analysis	
	sHR (95% C.I.)	*p* value	sHR (95% C.I.)	*p* value
Age	1 [0.96–1.05]	0.49		
Gender (male)	1.8 [1.02–3.16]	0.043	1.76 [1–3.09]	0.049
CRP at admission	0.99 [0.98–1.04]	0.33		
Increase of CRP > 30% in 5 days	1.43 [0.90–2.10]	0.43		
WBC at admission	0.98 [0.95–1.02]	0.34		
Increase of WBC > 30% in 5 days	3.10 [1.69–4.5]	0.001	3.25 [1.8–5.6]	0.0001
Eosinophil % at admission	1.42 [0.82–2.5]	0.183		
Ventilation days	1.04 [1.03–1.04]	0.042		
SAPS2 at admission	1.04 [1.02–1.05]	< 0.00001		
Chronic lung disease	0.96 [0.70–1.20]	0.55		
Hypertension	0.52 [0.30–1.64]	0.91		
Heart disease	0.91 [0.57–1.45]	0.69		
Shockable rhythm	3.03 [1.53–5.99]	0.0014		
Diabetes	0.87 [0.45–1.67]	0.68		
Obesity	1.42 [0.56–3.5]	0.63		
Kidney disease	0.91 [0.39–2.13]	0.83		
Cancer	1.68 [1.09–3.9]	0.02		
Pharyngeal yeast	1.44 [0.93–2.22]	0.29		
Pharyngeal bacteria	1.72 [1.03–2.87]	0.039	1.67 [1.01–2.76]	0.047

*Note:* For the multivariate analysis only the independently associated variables with sHR > 1 are reported.

**Table 4:** Micro-organisms found in respiratory secretions.

Causative micro-organisms	VAP *n* (% of identified)	Non-VAP (% of identified)
Not identified	2	6
Identified	7	10
MDR bacteria	4 (57.1%)	0
MRSA	3 (42.9%)	0
AMPc β lactamase producing *Escherichia coli*	1 (11.1%)	0
Not MDR bacteria	3 (42.9)	9 (90%)
*Enterococcus faecalis*	1 (14.3%)	1 (10%)
*MSSA*⁣^∗^	2 (28.6%)	1 (10%)
*Pseudomonas aeruginosa*	0	2 (20%)
*Enterobacter aerogenes*	0	1 (10%)
*Heamophilus influenzae*⁣^∗^	0	2 (20%)
*E. coli*⁣^∗^	0	2 (20%)
Virus	0	1 (10%)
*Influenza A H1N1*	0	1 (10%)
Total	9	16

⁣^∗^Bacteria susceptible to cefotaxime.

**Table 5:** Illustrate variations in VAP incidence and differences in MDRO involvement in studies.

Study	Population	Treatment	VAP incidence	Observations on MDROs
Scamperle et al.	Cardiac arrest patients	Topical and cefotaxime IV or ceftazidime IV	5.7%	Limited data
Francois et al.	OHCA with hypothermia	Amoxicillin-clavulanate IV	23%	Lower MDRO rates with IV antibiotics
Present study	OHCA with TM + SDD	SDD + cefotaxime IV	5.3%	Predominantly MDR pathogens in VAP

## Data Availability

The data that support the findings of this study will be made available upon reasonable request to the corresponding author. Due to patient confidentiality and ethical restrictions, the dataset is not publicly accessible. However, de-identified data can be shared upon approval from the institutional review board (IRB) and in compliance with applicable regulations.

## Nomenclature

TTM: Thermal treatment management
OHCA: Out-of-hospital cardiac arrest
SDD: Selective digestive decontamination
ICU: Intensive care unit
MDRO: Multidrug-resistant organisms
MRSA: Methicillin-resistant *Staphylococcus aureus*
SAPS: Simplified Acute Physiology Score
CFU: Colony-forming unit
VAP: Ventilator-associated pneumonia
WBC: White blood count
CRP: C-reactive protein
PCT: Procalcitonin
PCAS: Postcardiac arrest syndrome
EOP: Early-onset pneumonia
